# Systematic Review of Willingness to Pay for Health Insurance in Low and Middle Income Countries

**DOI:** 10.1371/journal.pone.0157470

**Published:** 2016-06-30

**Authors:** Shirin Nosratnejad, Arash Rashidian, David Mark Dror

**Affiliations:** 1 Iranian Center of Excellence in Health Services Management, School of Management and Medical Informatics, Tabriz University of Medical Sciences, Tabriz, Iran; 2 Tabriz Health Services Management Research Center, Department of Health Services Management, School of Management and Medical Informatics, Tabriz University of Medical Sciences, Tabriz, Iran; 3 Department of Health Management and Economics, School of Public Health, Tehran University of Medical Sciences, Tehran, Iran; 4 Knowledge Utilization Research Center, Tehran University of Medical Sciences, Tehran, Iran; 5 Micro Insurance Academy, New Delhi, India; 6 Former hon. Professor, Erasmus University Rotterdam, Rotterdam, Netherland; London School of Hygiene and Tropical Medicine, UNITED KINGDOM

## Abstract

**Objective:**

Access to healthcare is mostly contingent on out-of-pocket spending (OOPS) by health seekers, particularly in low- and middle-income countries (LMICs). This would require many LMICs to raise enough funds to achieve universal health insurance coverage. But, are individuals or households willing to pay for health insurance, and how much? What factors positively affect WTP for health insurance? We wanted to examine the evidence for this, through a review of the literature.

**Methods:**

We systematically searched databases up to February 2016 and included studies of individual or household WTP for health insurance. Two authors appraised the identified studies. We estimated the WTP as a percentage of GDP per capita, and adjusted net national income per capita of each country. We used meta-analysis to calculate WTP means and confidence intervals, and vote-counting to identify the variables that more often affected WTP.

**Result:**

16 studies (21 articles) from ten countries met the inclusion criteria. The mean WTP of individuals was 1.18% of GDP per capita and 1.39% of adjusted net national income per capita. The corresponding figures for households were 1.82% and 2.16%, respectively. Increases in family size, education level and income were consistently correlated with higher WTP for insurance, and increases in age were correlated with reduced WTP.

**Conclusions:**

The WTP for healthcare insurance among rural households in LMICs was just below 2% of the GPD per capita. The findings demonstrate that in moving towards universal health coverage in LMICs, governments should not rely on households' premiums as a major financing source and should increase their fiscal capacity for an equitable health care system using other sources.

## Introduction

The interest in Willingness To Pay (WTP) for health insurance arises in settings where on the one hand access to healthcare is mostly contingent on Out-Of-Pocket Spending (OOPS) by health seekers, and on the other hand health insurance schemes must know upfront how much they could charge as premium, to ensure their financial sustainability. This is particularly relevant in Low- and Middle-Income Countries (LMICs); by one estimate, only 5–10% of population in sub-Saharan Africa and South Asia are covered by social healthcare schemes that are funded by the state [1], and in many middle-income countries the effective cost coverage of mandatory health insurance schemes ranges from 20 to 60% [1], leaving even insured persons exposed to considerable OOP.

In 2012 the UN General Assembly, in the framework of the Resolution on ‘universal health coverage’, called upon Member States “*to ensure that health financing systems evolve so as to avoid significant direct payments at the point of delivery and include a method for prepayment of financial contributions for health care and services as well as a mechanism to pool risks among the population in order to avoid catastrophic health-care expenditure and impoverishment of individuals as a result of seeking the care needed*” [2]. This framing would require many LMICs to raise enough funds to achieve universal health insurance coverage. This poses a number of questions: are individuals or households willing to pay for health insurance, and how much? What factors positively affect WTP for health insurance? And is the role of governments obviated when private health insurance is offered? This study focuses on reviewing the evidence about WTP for social or other non-commercial health insurance (including community-based mutual health insurance) with the view to offer policymakers an estimate of the resources that can be generated from uninsured population segments were health insurance offered, based on the evidence from pilot experiments reported in the existing literature from low- and middle income countries.

There are two approaches to estimating WTP: “Revealed Preferences” (RP) [3], and “Stated Preferences” (SP)[4]. RP is a predictive, modeling approach to WTP, based on studying actual purchasing behavior of products from which we wish to estimate the WTP for the product for which no purchasing information exists and that we are interested in. Unfortunately, we could not find any published study using the RP method to estimate WTP for health insurance in LMIC.

The alternative option, SP, is to ask people what they would be willing to pay for insurance cover that they do not yet have, and that is perhaps not even on the market. Several SP methods evolved to value non-market goods. One of the most frequently used, called “Contingent Valuation” (CV)[3, 4] consists of using survey methods to present respondents with hypothetical scenarios about an intervention under evaluation (or insurance product in our case). Respondents are required to think about the contingency (or feasibility) of an actual market for the benefits, and state the maximum they would be willing to pay for them. This method assumes that prospective clients are given a detailed description of the product for which they are asked how much they would be willing to pay. However, if the price determines the product, and is not yet known, how then is it possible to guarantee that the described product would actually be the one delivered?

WTP is presumably mediated by ability to pay and by individual and cultural aspects that determine how the utility of insurance is perceived. We wanted to examine the evidence for this, through a review of the literature. Different authors have used different anchors to measure the WTP against (e.g. income, disposable income, food expenditure etc.). In the interest of comparison across countries and different socioeconomic contexts, we propose to assess WTP levels against per capita GDP.

This article is structured as follows: section 2 contains methodological notes. Results of the review are presented in Section 3, and a discussion and conclusions in the 4^th^ and last section.

## Methods

### Information Sources

The literature search (conducted in February 2016) included academic and gray literature. The academic thematic databases included Pubmed, Science Direct and Scopus and search engine of Google scholar. The gray literature was searched manually, through citation tracking.

### Search Strategy

Search strategies for electronic databases were developed by one of the authors (SN) and peer-reviewed by other authors. One of the authors conducted the search. We used “willingness to pay" and "insurance” as the key terms, with no limits for study designs and date of publication, while limiting the searches to English language for practical reasons. The search identified studies published until 2016. The complete Medline/PubMed search strategy is available as Supporting Information (S1 Text).

### Eligibility Criteria (Inclusion/Exclusion)

Primary studies that assessed households or individuals' WTP for health insurance in LMIC were eligible for inclusion. Studies were considered eligible for inclusion if:

1. Collected data from individuals or households living in one or more LMIC.
2. Estimated individuals or households’ WTP for health insurance using monetary value or as a percentage of income
3. Reported data on WTP for social, national or community health insurance.

These selection criteria meant that studies that focused solely on insurance of other risks, or health insurance that was based on individual risk rating and without any aspect of community rating for the purpose of defining the level of payment requested from the individual (e.g. private actuarial insurance) were not eligible for inclusion. Finally, studies that assessed WTP for certain health care services (e.g. insurance for dental care or complementary inpatient insurance) were not eligible for inclusion.

A priori, we considered any previously published systematic review studies of WTP for health insurance as eligible for inclusion in this review, if they answered the same questions. No other article adopted the exact same inclusion criteria; we found one study on the same topic which did not expressly exclude private health insurance but was also focused in LMIC, where private health insurance is rare[5].Those articles retained 13 articles within the date range from 1996 to 2010, of which 12 are also included in this systematic review; this review includes 11 additional studies (for a total of 23 articles). And its analysis followed a different method[5]. Hence, we do not have a previous study to which we can compare our results.

### Data Extraction

One author extracted data from included studies and entered them into the data extraction sheet that was then checked by another author for accuracy and completeness. Data extracted included location and year of study, sample size, response rate, target population, household size, data collection method, variables that significantly affected WTP, and WTP values (per individual and per household) reported in the study. Additionally, we collected data on country specific Gross Domestic Product (GDP) per capita, net national income and population in the year of the study from the World Bank's list of indicators[6]

### Critical appraisal of studies: quality assurance process

Quality assurance was maintained in four stages: (1) All duplicates were eliminated, usingEnd-Note software version 7 (http://endnote.com/) (2). The titles and the abstracts of identified papers were screened, and those that were obviously unrelated to our review were excluded. (3) Two authors assessed the full texts of the retained articles and any discrepancies between them resolved through discussion. (4) The critical appraisal of included studies was done by the STROBE tool [7] and in this stage we also considered some methodological recommendations in appraisal of studies (S1 Table) [8, 9].

### Data Analysis and Synthesis

We used two analytical approaches

1. A meta-analysis synthesis to assess WTP
2. A vote counting to identify the main attributes that affect WTP.

A meta-analytic synthesis of the studies, conducted with sub-topics as follows:

#### Estimating effect size

The included studies in this meta-analysis reported WTP values. We used the following way to convert these values into the effect size: the values were transformed to annual values and converted to US$ using the exchange rate for the study year, as reported in the study. If not reported, we used the International Monetary Fund (IMF) values [10]. In the next step, we estimated the WTP of individuals or households as a percentage of GDP per capita and as a percentage of adjusted net national income per capita of each country.

#### Estimating standard error of the effect size

Standard error (SE) of the effect size was estimated from the SE of the WTP values by applying similar transformation used for estimating the effect size. Some authors reported the 95 percent confidence intervals instead of the SE. For these studies we first computed the SE of the values and then computed the SE of the effect size by using the following way.

Some authors did not report the SE, CI (Class Interval). Therefore, we could not estimate the SE of the effect size for these studies [11–13]

#### Weights

The standard practice in meta-analysis is to apply weights proportional to the variances of the effect size for estimating the summary effect. In this meta-analysis we applied the SEs of the effect size as a weight.

#### Estimating summary effect

When a WTP values were computed in the same way by all studies, the summary effect was obtained by averaging the effect sizes, after applying weights. We used fixed and random effect meta-analysis techniques to calculate summary effect, based on the level of heterogeneity between the included studies.

A descriptive vote-counting approach conducted with sub-topics as follows [14, 15]:

- We identified Different objective variables that influenced willingness to pay as assessed in regression analyses reported in the included studies
- We categorized the variables by those that had significant positive, significant negative or non-significant effects on WTP in their respective study sample.
- Each study casts a “vote” in support of the above relationships and the numbers of the votes were counted
- If a variable obtained a minimum of three votes, we considered that variable likely to affect the WTP for health insurance.

We follow the PRISMA approach in performing this systematic review, and we attached the PRISMA form as appendix (S1 Appendix).

## Results

### Search result

**Fig 1:**
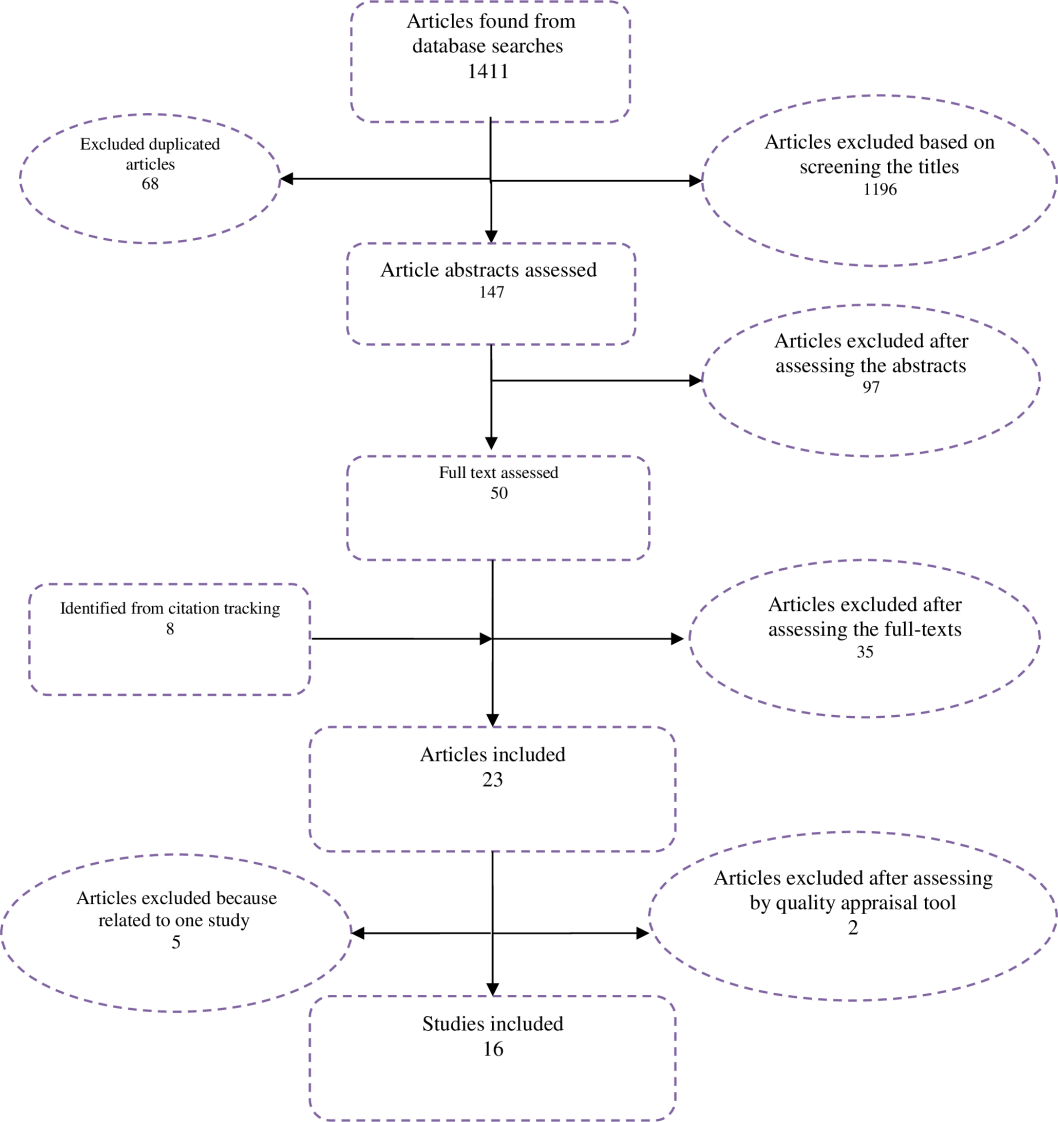
Article flowchart for studies of WTP for insurance demonstrating the studies identified assessed and included in the systematic review.

Our academic database searches yielded 1411 hits and the gray literature searches another 8 articles. After screenings of the titles and abstracts, we kept58 studies for full-text review (Fig 1), and thereafter retained 23 articles for detailed review [11–13, 16–35]. Of these, two articles [26] and [20] were excluded after quality assessment.

The remaining 21 articles relate to 16 distinct studies in the following ten countries: In Asia: Bangladesh [34], China [27], India [18, 19, 28, 33], Iran [21, 32] and Vietnam [12]. In Sub- Saharan African: Burkina Faso [16], Cameroon [22, 30], Ethiopia [13], Ghana [11], and Nigeria [17, 29]. The studies altogether had surveyed about 1050 households plus about 9857 individuals.

Consequently, in Table 1 we included 17 studies (as the study of the scheme in Burkina Faso is considered by reference to two articles by Dong et al (one relating to households and the other to individual WTP in the same scheme). In Table 2, we only listed the 12 out of 16 distinct studies which had adequate data for Meta-analysis. And in Table 3 we list only 16 studies, since the variables under consideration allowed considering Burkina Faso as a single study.

**Table 1:** Summary of articles conducted in different countries on willingness to pay for health insurance.

	Author (year)	Country	Year	Population	Household or individual	Sample size	Measurement method of WTP	WTP as a percentage of GDP per capita	WTP as a percentage of net national income per capita
**1**	**Asenso-Okyereet al., 1997 [11]**	Ghana	1992	Rural and urban	Household	306	bidding game	8.07	9.13
					individual			1.61	1.83
**2**	**Mathiyazhagan et al., 1998 [19]**	India	1995	Rural	Household	918	open-ended questions	0.84	1.01
**3**	**Barnighausen et al., 2007**	China	2000	Urban	Individual	651	payment card	6.07	7
**4**	**Dong et al., 2003 [16]**	Burkina Faso	2001	Rural and urban	Household	705	bidding game	5.86	6.28
**5**	**Dong et al., 2004 [23]**	Burkina Faso	2001	Rural and urban	Individual	2414	bidding game	2.15	2.3
							Take- it- or leave—it		
**6**	**Asgary et al., 2004 [21]**	Iran	2001	Rural	Household	2139	bidding game	1.9	2.82
**7**	**Binam et al., 2004 [22]**	Cameroon	2002	Rural	Individual	471	bidding game	2.23	2.61
**8**	**Lofgren et al., 2008 [12]**	Vietnam	2004	Rural	Household	2063	Biding game	1.85	2.36
					individual		open- ended question	0.41	0.53
**9**	**Dror et al., 2007 [28]**	India	2005	Rural and urban	Household	3024	bidding game	1.82	2.12
					individual			0.39	0.46
**10**	**Ataguba et al., 2008 [29]**	Nigeria	2007	Rural	Household	309	payment card,	2.15	3.06
					Individual		double bounded dichotomous choice	0.35	0.51
							open- ended question		
**11**	**Onwujekwe et al., 2010 [17]**	Nigeria	2007	Rural and urban	Individual	3070	bidding game	2.32	3.3
**12**	**Donfouet et al., 2010–2011 [30, 31]**	Cameroon	2009	Rural	Individual	399	double bounded dichotomous choice	1.98	2.52
							open -ended question		
**13**	**Ghosh et al., 2011 [De 18]**	India	2010	Urban	Individual	1502	double bounded dichotomous choice	_	_
**14**	**AbayAsfaw et al., 2004 [13]**	Ethiopia	2001	Rural	Household	550	double bounded dichotomous choice	12.2	-
**15**	**Kumar et al., 2015 [33]**	India	2008	Urban	Individual	500	open -ended question	0.45	0.54
**16**	**Sayem Ahmed et al., 2016 [34]**	Bangladesh	2011	Urban	Individual	557	bidding game	1.63	2.05
**17**	**Nosratnejad et al., 2014 [32]**	Iran	2016	Urban	Individual	300	double bounded dichotomous choice	0.99	1.33

**Table 2:** WTPs as a percentage of GDP and net national income: the results of meta-analysis of the included studies.

Variable	Method	Pooled estimate	95% confidence interval	P-value	N of studies [ref]
**WTP household (% GDP per capita)**	Fixed effect	1.82	1.5–2.15	0.000	4 [16, 19, 21, 29]
**WTP Household (% net national income per capita)**	Fixed effect	2.16	1.83–2.50	0.000	4 [16, 19, 21, 29]
**WTP of individual (% GDP per capita)**	fixed effect	1.18	1.13–1.24	.0.000	10 [16, 17, 22, 27–30, 32–34]
**WTP of individual (% net national income per capita)**	fixed effect	1.39	1.34–1.44	0.000	10 [16, 17, 22, 27–30, 32–34]

**Table 3:** Variables that influenced WTP in the included studies^$^.

	Demographic					Socio- economic				Health service	Perceived need			Insurance related
	Male gender	Age	Married	Family size	Having children under 5	Income	Education	Employment statues	Living in rural areas	Distance to preferred health facility	Past health expenditure	Incidence of hospitalization	Poor health status	Insurance experience
**Asenso-Okyere et al., 1997**	^↓↓^	^**↑**^	^**↑**^	^**↑↑**^	^**_**^	^↓↓^	^↓↓^	^**↓**^	^**↓**^	^**↑**^	^↓↓^	** -**	**-**	^**↓**^
**Mathiyazhagan et al., 1998**	**-**	^**↓**^	^**- **^	^**↑↑**^	**-**	^**↑↑**^	^**-**^	^**↑↑**^	**-**	^**↑↑**^	**-**	^**↑↑**^	^**↑↑**^	**-**
**Barnighausen et al., 2007**	^↓↓^	^↓↓^	** -**	**-**	**-**	^**↑↑**^	^**↑**^	^↓↓^	**-**	**-**	^**↑↑**^	** -**	**-**	**-**
**Dong et al., 2003**	^**↑↑**^	^↓↓^	^**↑**^	^**↑**^	^**↑**^	^**↑↑**^	^**↑↑**^	**-**	^**↑↑**^	^↓↓^	^**↑↑**^	^** **^**-**	^**↓**^	**-**
**Asgary et al., 2004**	^**↑**^	^**↑↑**^	** -**	^**↓**^	**-**	^**↑**^	^**↑↑**^	**-**	^↓↓^	^**↑↑**^	**-**	**-**	**-**	^**↓**^
**Binam et al., 2004**	^**↑↑**^	^**↑**^	^**↓**^	^**↑↑**^	**-**	^**↑↑**^	^**↑**^	**-**	**-**	**-**	**-**	^**↑↑**^	^**↑↑**^	**-**
**Lofgren et al., 2008**	^**↑**^	^↓↓^	** -**	^**↑↑**^	^**↑**^	^**↑↑**^	^**↑↑**^	^**↓**^	**-**	**-**	**-**	**-**	**-**	^**↑**^
**Dror et al., 2007**	^**↑↑**^	^↓↓^	** -**	^**↑↑**^	^**↑**^	^**↑↑**^	^**↑↑**^	**-**	^**$ $**^	^↓↓^	**-**	^**↑↑**^	**-**	^**↑↑**^
**Ataguba et al., 2008**	^**↑↑**^	^**↓**^	^** **^**-**	**-**	**-**	^**-**^	^**↑**^	**-**	**-**	^**↑↑**^	**-**	**-**	^**↑**^	**-**
**Onwujekwe et al., 2010**	^**↑↑**^	**-**	** -**	^↓↓^	**-**	**-**	^**↑↑**^	**-**	^↓↓^	**-**	**-**	**-**	**-**	**-**
**Donfouet et al., 2010–2011**	^**↑**^	^↓↓^	^** **^**-**	^**↑**^	**-**	^**↑↑**^	^**↑**^	^↓↓^	**-**	**-**	**-**	**-**	^**↑**^	**-**
**Ghosh et al., 2011**	**-**	^**↓**^	^** **^**-**	**-**	**-**	^**↑↑**^	^**↑**^	**-**	**-**	**-**	**-**	^**↑↑**^	^**↑↑**^	**-**
**AbayAsfaw et al., 2004**	^**↑**^	^**↓**^	**-**	^**↑↑**^		^**↑↑**^	^**↑↑**^						^**↑**^	
**Kumar et al.,2014**		^**↓**^	^**↑**^	^**↑**^	**-**	^**-**^	^**↑↑**^	**-**	**-**	**-**	**-**	**-**	**-**	**-**
**Sayem Ahmed et al., 2016**	^**↑**^	^↓↓^	^**↑**^	^**↑↑**^	**-**	^**↑↑**^	^**↑↑**^	**-**	**-**	**-**	**-**	**-**	^**↑**^	**-**
**Nosratnejad et al., 2014**	^**↑**^	^**↑**^	^**-**^	^**↑↑**^	^**↑**^	^**↑↑**^	^**↑↑**^	^**↑↑**^	**-**	**-**	**-**	^**↑**^	^**↓**^	^**↑↑**^

^$^ Variables that mentioned in more than 3 studies are indicated in the Table.

^$ $^ The study had conflicting results (i.e. different rural and urban regions provided varying WTP estimates).

^↑↑^ the effect of variable is positive and significant.

^↓↓^ the effect of variable is negative and significant.

^↓^ the effect of variable is negative and non- significant.

^↑^ the effect of variable is positive and non-significant.

### Quality Assessment result

Details of the quality of studies are presented in appendix (S2 Appendix). The overall methodological quality of included empirical studies was good, with more than half of studies meeting all criteria. Two studies [20, 26] that did not rank suitably according to this appraisal were excluded from further analysis.

### Studies characteristics

Table 1 contains information on study characteristics.

The sample sizes of the studies varied greatly, from 300 to 3,070 individuals. Seven studies were conducted in rural settings[12, 13, 19, 21, 22, 29, 30], four studies on rural and urban residents [11, 16, 17, 28], one study on urban residents [32], three studies on urban informal sector employees [27, 33, 34] and one study on the urban poor [18]. Most of the studies involved face-to-face interviews with the respondents. All of the identified studies used contingent valuation for WTP estimation: bidding game (eight), open ended (five), double-bounded (five), payment cards (two) and single bounded (one) methods, including four studies that used more than one method.

### WTP for health insurance

We were able to calculate the WTP as percentages of GDP and net national income per capita for all except one included study; that one exception estimated the WTP only as a percentage of household income and did not report monetary values[18]. Four studies that measured household WTP provided enough data to enable us to calculate the corresponding individual WTP[11, 12, 19, 29].

**Fig 2:**
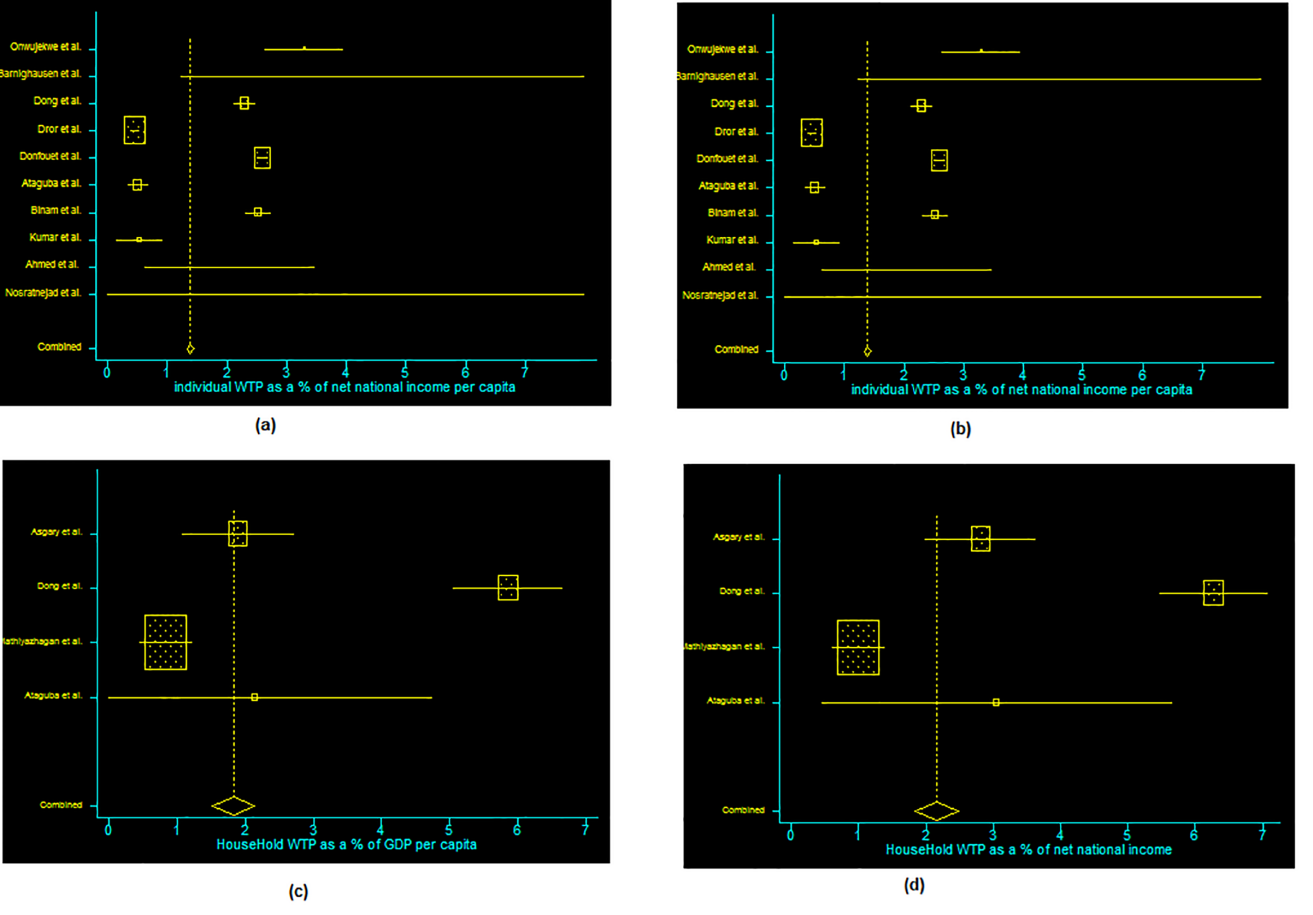
**The reported Willingness to pay for health insurance in different studies, and the overall estimated WTP of individual as a % of GDP (a), and as a % of net national income (b), WTP for household as a % of GDP (c) and as a % of net national income (d)**.

Out of 15 studies, ten studies provided enough data to conduct a meta-analysis of WTP at individual level, using national GDP data: 1.18% of GDP per capita (95% CI: 1.13–1.24) and 1.39% of adjusted net national income per capita (95% CI: 1.34–1.44). Out of five studies, four studies provided enough data to conduct a meta-analysis of WTP at household level: 1.82% of GDP per capita (95% CI: 1.5–2.15) and 2.16% of adjusted net national income per capita (95% CI: 1.83–2.50)(Table 2) (Fig 2). All the meta-analysis resulted in small p-values suggesting that the findings were unlikely to have occurred due to chance, noting the studies were conducted in different times and different countries.

We repeated the analyses after excluding the studies conducted in urban areas that reported substantially higher WTPs, but this did not affect the meta-analysis results.

### Variables that influenced WTP for healthcare insurance

We categorized all objective variables that were assessed for their influence on WTP for health insurance into five groups, as follows:

- Demographic determinants. *‘*Marital status’ and ‘having a child under five in the household’ did not affect willingness to pay. ‘Male gender’ (5 to 2) and a larger ‘family size’ (8 to 1) were linked with higher willingness to pay, while ‘older age’ (6 to 1) was correlated with lower willingness to pay for health insurance.
- Socioeconomic determinants. The ‘income level’ (11 to 1) and ‘education level’ (9 to 1) higher than primary education or highest grade educations were linked with an increased willingness to pay. We found equivocal evidence of the potential effects of ‘living in rural areas’ and ‘employment statuses on willingness to pay.
- Health services determinants. The results for effects of ‘distance to the nearest or preferred hospital’ on willingness to pay were equivocal.
- Perceived needs determinants. A ‘past experience of hospitalization’ (4 to 0) and a ‘perceived poor health status’ (3 to 0) were linked with a higher willingness to pay. The findings for ‘past health care expenditure’ were equivocal.
- Insurance related variables. Effects of ‘experience of having insurance’ (2 to 0) were linked with a higher willingness to pay (See “S2 Text” for more details). Fourteen variables were included in at least three studies each and were included in vote-counting analyses.

## Discussion

Our study is the first systematic review of WTP of individuals or households for health insurance in LMICs. We identified 21 articles (16 distinct studies) assessing households or individuals’ WTP. Our study had two main objectives that we discuss in turn: identifying the variables that consistently affected WTP for insurance in different settings, and a transferrable estimation of the level of WTP in LMICs (as a percentage of GDP or net income per capita).

Different demand theories explain decisions to purchase health insurance [36, 37]. According to these theories, factors such as the price of insurance, degrees of risk aversion, household ability to pay and income, the magnitude of expected loss from illness (i.e. user fees), the probability of illness, and the expected performance of the insurer (the service coverage and the amount of pay-off) might affect the decision to insure and the WTP for insurance. While these theories are generally unequivocal in their expectation of the impact of different variables on WTP for insurance, our findings suggested that only a limited set of empirical variables corresponding to the theory driven factors consistently affected the WTP for insurance. This finding upholds the newest study on theory of demand for health insurance [38].

Our findings suggest that increase in family size, education level, income, past hospitalization and perceived poor health status were consistently correlated with higher WTP for insurance. Increased household income enables the households to purchase more health insurance and increase the demand for health insurance [39]. A previous study estimated that a 1% increase in national income might lead to a 0.9% increase in health spending after controlling for other factors [40]. We also noted that increases in age were correlated with reduced WTP. This seems surprising as it is widely assumed that older people incur higher health expenditure [41]; however, a recent review noted that the effects of older age on health care were lesser than argued in the past [40]. Another recent study suggested that older people might have less capacity to pay for needed health care [36], and to pay for health insurance. These different views suggest that our finding of lower WTP for health insurance among older people requires further study.

Other factors may also affect WTP. For example, when the concept / value proposition of “insurance” is unclear, or when households consider their ability to avail health care as slim (e.g. because the service provider is too far away, or because of previous negative experiences), they may report a lower WTP [42]. Similarly, when their past experience suggests improvements in health status because of the service (even if it is far away) they may be willing to pay more for the insurance. Hence it may be difficult to separate the impact of hypothetical scenario or access barriers on WTP. We did not observe a consistent picture in different studies.

The results of the meta-analysis suggest that WTP amounted to just below two per cent of GDP per capita per household per year. A lower figure was obtained for individuals' WTP. WTP, by definition, is the maximum amount people are willing to spend to acquire a service [17]. Despite this theoretical underpinning of the WTP concept, we should interpret the findings of this review with care. Many of the studies included in the meta-analysis originated from rural areas of low-income countries. Rural residents may have a limited understanding of insurance and risk management, and this may affect their WTP. Also as they generally have a lower socioeconomic status than the rest of the country, they may have a lower WTP. The six studies that included urban residents reported WTP values higher than other studies, although the three studies conducted after 2012 reported lower WTP values as a proportion of GDP per capita [32–34].

In many LMICs, households with a lower socioeconomic status spend substantial amounts out-of-pocket on essential needs for health care [43]. Hence they may be willing to pay for health insurance, if they have a positive expectation that health insurance would provide effective social protection against health-induced poverty and catastrophic expenditures [44]. Others [12, 29] have suggested that the surveys may underestimate WTP; one way this was explained has been that households facing high out-of-pocket medical expenses and risk catastrophic health care expenditure may state lower WTP for insurance [12]. Another argument has been that in some LMICs people may have a higher WTP if the payment is in kind and via commodities, but lower when WTP must be made as cash payment [29].

Most of the included studies used bidding game methods for the estimation of WTP. While the bidding game is simple to conduct, it has some inherent disadvantages. The initial anchor (or 'price' offered) in a bidding game may influence the WTP estimate to be higher than that identified using the other contingent valuation methods [24].

This is the first study proposing to express WTP estimates as a percentage of GDP per capita of the respective country, or as a percentage of national net income per capita. The idea is that such estimates might provide a powerful tool for policymakers in planning revenue generation through a national social or other broad format of non-commercial health insurance program. Estimating a rough figure that is linked to the general economic status of a country should only act as a starting point where local specific data is not available. While many countries lack national representative data to enable them to assess average WTP for health insurance, the review findings might be preferable than basing these estimates on small samples in a limited area of a country.

There might be other benefits for having such estimates. While LMICs may use past experiences of other countries and the advice of donors and international organizations for setting benefit packages, they often face the challenges of having few financial resources to cover the costs. Estimating the WTP as a percentage of GDP can help foresee what resources might be available from households, resources that are often untapped and if generated could sustainably provide an affordable benefit package. Our overall estimate of WTP for health insurance should be interpreted as a likely minimum value, which could increase over time when the social or community-based health insurance delivers compelling results. That said, households with a low socioeconomic status would perhaps find it very difficult to pay the same level of national community rated contribution.

## Conclusions

This systematic review of the literature on willingness to pay for social and community-based (non-commercial) health insurance in low- and middle income countries has shown that following the classical theoretical precepts on demand for health insurance is not very helpful, since only some but not all “assumed predictors” are actually borne out by the empirical evidence. From our study we conclude that we cannot find sufficient evidence linking WTP to factors such as the price of insurance, degrees of risk aversion, the magnitude of expected loss from illness or the probability of illness. Our findings point that factors which were consistently correlated with higher WTP for insurance include family size, education level, income, past hospitalization and perceived poor health status. We also noted that people of elderly age were correlated with reduced WTP, which is probably due to lower capacity to pay rather than lower perception of risk. We also conclude that low awareness to the value proposition of insurance is associated with a lower WTP.

The results of our meta-analysis suggest that WTP amounted to just below two per cent of GDP per capita per household per year, with a lower figure obtained for individuals' WTP. Considering that many of the studies included in the meta-analysis reported data originating from rural areas of low-income countries, and considering the cumulative effect of a limited understanding of insurance and a lower socioeconomic status among rural populations, one might treat the results as a minimal expression of WTP.

The findings might provide a general guide for national policy makers as well as donors in deciding the amount they should provide over and above the resources that households might be required and willing to contribute toward the provision of social or community-based health insurance.

## Supporting Information

S1 Appendix The PRISMA checklist. (DOC)

S2 Appendix Quality appraisal of included studies. (XLSX)

S1 Table Methodological recommendations in appraisal of studies. (DOCX)

S1 Text The complete PubMed search strategy. (DOCX)

S2 Text The full list of variables that were assessed in primary studies for their influence on willingness to pay for health insurance. (DOC)
