# Supplementary material for: Systematic Review of Willingness to Pay for Health Insurance in Low and Middle Income Countries
Source: PLoS One. 2016 Jun 30;11(6):e0157470. doi: 10.1371/journal.pone.0157470 (PMC4928775; doi:10.1371/journal.pone.0157470)
Supplement: S1 Table — (DOCX) [file pone.0157470.s003.docx]

**Support Information 2 (SI 2):**methodological recommendations in appraisal of studies

| **Question** | **yes** | **no** | **Un clear** |
| --- | --- | --- | --- |
| Does a detailed description of goods or services in question offered to the respondents? |  |  |  |
| Does the information and attributes expressed in goods or services scenarios is obtained from user or key informant assessments (e.g. focus groups, Delphi panels, interviews etc)? |  |  |  |
| Was there a pilot study conducted to assess the survey tool/design? |  |  |  |
| Does the survey involve face to face interviews? |  |  |  |
| Was the usual payment amount (e.g. existing insurance premiums) are used as a payment vehicle in study? |  |  |  |
