# Supplementary material for: Systematic Review of Willingness to Pay for Health Insurance in Low and Middle Income Countries
Source: PLoS One. 2016 Jun 30;11(6):e0157470. doi: 10.1371/journal.pone.0157470 (PMC4928775; doi:10.1371/journal.pone.0157470)
Supplement: S1 Text — (DOCX) [file pone.0157470.s004.docx]

**S1 Text:** the complete PubMed search strategy

#1 Search willingness to pay OR willingness- to-pay OR WTP OR demand OR contingent valuation* Field: Title/Abstract,

Limits: humans /English language

#2 Search insurance Field: Title/Abstract,

Limits: humans / English language

Search #1 AND #2 Field: Title/Abstract, Limits: Humans / English language
