# Supplementary material for: Systematic Review of Willingness to Pay for Health Insurance in Low and Middle Income Countries
Source: PLoS One. 2016 Jun 30;11(6):e0157470. doi: 10.1371/journal.pone.0157470 (PMC4928775; doi:10.1371/journal.pone.0157470)
Supplement: S2 Text — (DOC) [file pone.0157470.s005.doc]

| S2 Text: The full list of variables that were assessed in primary studies for their influence on willingness to pay for health insurance   - *Demographic determinants*   age, gender, marital status, having a child under five or an elderly of 65 years or more in the household, family size, religion;   - *Socio-economic determinants*   education, living in urban or rural areas, place of living, household living standards, immigration status, occupation status, income level, total household expenditure, household wealth;   - *Health service determinants*   distance from or travel time to health facilities, waiting time for health care, access to physicians, availability of basic drugs at the health center;   - *Perceived need determinants*   having a household member with a chronic disease, having a sick person in the household, health status, number of working days lost due to illness, hospital care utilization, out-of-pocket health care expenditure;   - *Insurance-related determinants*   involvement in policy making, having insurance experience, knowledge about health insurance, insurance premium |
| --- |
